# Antibiotic resistance among ICU patients during the COVID-19 pandemic and its associated factors: a retrospective study using electronic medical records in two Vietnamese hospitals

**DOI:** 10.1017/S0950268825100307

**Published:** 2025-07-17

**Authors:** Dang-An Do, Vu-Minh Duy-Nguyen, Thi-Hang Nguyen, Dinh-Thanh-Son Le, Huy-Ngoc Nguyen, Duc-Nhu Dang

**Affiliations:** 1 Department of Community and Global Health, The University of Tokyo, Tokyo, Japan; 2 Department of Infectious Diseases, Institute of Science Tokyo, Tokyo, Japan; 3 Military Hospital 175, Ho Chi Minh City, Vietnam; 4 Industrial University of Ho Chi Minh City, Ho Chi Minh City, Vietnam; 5 Division of Training and Scientific Research, Thang Long Institute of Medical and Pharmaceutical Research, Hanoi, Vietnam; 6 Phu Tho Provincial General Hospital, Phu Tho, Vietnam; 7 Phu Tho Provincial Health Department, Phu Tho, Vietnam; 8 Faculty of Public Health, University of Medicine and Pharmacy, Vietnam National University, Hanoi, Vietnam

**Keywords:** antimicrobial resistance (AMR), antimicrobial stewardship (AMS), antimicrobial susceptibility testing (AST), COVID-19, infection prevention and control (IPC), medical informatics

## Abstract

Antimicrobial resistance (AMR) in intensive care units (ICUs) is a critical issue, which has been exacerbated by the coronavirus disease 2019 (COVID-19) pandemic. This study investigated AMR prevalence and its associated factors among ICU patients in two Vietnamese hospitals from January 2020 to June 2022. Electronic medical records of 1,296 patients with 2,432 non-duplicate bacterial isolates were collected in Phu Tho Hospital (Northern, rural, non-COVID-19 treatment) and 175 Hospital (Southern, urban, COVID-19 treatment centre). Antibiotic susceptibility testing was conducted using VITEK2, BD Phoenix 100, and disk diffusion methods. Logistic regression with 1,000 bootstrap resampling and cross-validation was used to examine factors linked to AMR. Results revealed *Acinetobacter spp.* (27.5%) as leading strains in Phu Tho Hospital, while *Klebsiella spp.* (28.0%) predominated in 175 Hospital, except during 2021when *Acinetobacter spp.* reached the peak. Alarmingly, *Acinetobacter spp., Klebsiella spp., and Pseudomonas aeruginosa* demonstrated the highest AMR rates and multidrug resistance rates (83.8%–95.8%) in both hospitals. Resistance to cephalosporins, carbapenems, and fluoroquinolones ranged from 75% to 100%. Significant associated factors included age, sex, location, initial admission diagnosis, and bacterial isolation month. This study highlights the urgent need for controlling AMR in ICUs during the pandemic.

## Introduction

Antimicrobial resistance (AMR) is a global health threat with projections of over eight million annual deaths associated with AMR by 2050 [1]. Six strains of bacteria, including *Escherichia coli (E. coli), Streptococcus pneumoniae, Staphylococcus aureus (S. aureus), Klebsiella pneumoniae, Acinetobacter baumannii (A. baumannii), and Pseudomonas aeruginosa (P. aeruginosa)*, account for more than 80% of AMR-attributed mortality worldwide [1]. Intensive care units (ICUs) represent high-risk settings, where AMR infections contribute to nearly 80% mortality rates [2] and 50% of hospital-acquired infections (HAI) [3]. The coronavirus disease 2019 (COVID-19) pandemic leveraged AMR challenges when antibiotic use was reported in 75% of admissions and bacterial AMR was found in 60.8% of infections among COVID-19 patients [4].

In Vietnamese hospital ICUs, studies reported alarming AMR rates and multidrug resistance (MDR) [5–7], particularly among gram-negative bacteria, with 30%–70% resistance to third- and fourth-generation cephalosporins, aminoglycosides, and fluoroquinolones [6]. Similar trends in low-and middle-income countries (LMICs), such as India, China, and Pakistan, highlight the pandemic’s role in exacerbating AMR through antibiotic overuse and overwhelmed healthcare systems [8].

While high-income countries increasingly applied electronic medical records (EMRs) and Machine Learning (ML) to predict AMR patterns, optimize antibiotic use, and reduce AMR rates in hospital settings [9], LMICs like Vietnam face implementation limitations [10]. Few hospitals integrate antibiotic susceptibility tests (AST) into EMRs, challenging real-time and systematic AMR surveillance. This study was conducted in ICUs of two Vietnamese hospitals (Phu Tho Hospital, 175 Hospital), aiming to determine the AMR prevalence among five high-priority bacterial pathogens *(E. coli, S. aureus, Klebsiella species, Acinetobacter species, P. aeruginosa)* and identify its associated factors during the COVID-19 pandemic.

## Methods

### Study design and settings

This retrospective cross-sectional study analysed EMRs from ICU patients at the Phu Tho Provincial General Hospital (Phu Tho Hospital) and the Military Hospital 175 (175 Hospital). These hospitals were selected purposively [11], in which Phu Tho Hospital was in the Northern, rural, and mountainous region, meanwhile, 175 Hospital was in the Southern, urban, and densely populated region. In the 2020s, the gross regional domestic product *per capita* in Phu Tho Province and Ho Chi Minh City was 2,184 and 6,405 USD, respectively [12, 13]. While 175 Hospital serves military personnel, it also treated civilians, especially during the COVID-19 pandemic [14, 15]. Phu Tho Hospital did not treat the COVID-19 patients during the period of study (2020–2022).

EMRs of patients aged ≥18 years upon admission with positive bacterial cultures in the ICUs were collected from 1 January 2020, to 30 June 2022, covering four COVID-19 waves in Vietnam [16]). Wave 1 (January–July 2020, imported cases, no deaths); Wave 2 (July–December 2020, the first epidemic wave with local transmission, 35 deaths); Wave 3 (January–March 2021, imported Alpha variant, no fatalities); and Wave 4 (April 2021–March 2022, Delta variants, approximately 32,000 deaths [16]).

Viral infections and fungal isolates were excluded, duplicate bacterial isolates (defined as those of the same species and susceptibility profiles from the same patient at admission) were removed to ensure unique observation and prevent overestimations. Extracted data included patient demographics (*e.g.*, age, sex), clinical history (initial diagnosis, time of bacterial isolation), and AST results.

### Sample size

From 3,965 AST samples recorded over two and a half years, 2,432 non-duplicated bacterial samples were analysed after excluding fungal samples and duplicates, including 856 from Phu Tho Hospital and 1,576 from 175 Hospital. These samples originated from 1,625 EMRs of 1,296 ICU patients (600 from Phu Tho Hospital and 696 from 175 Hospital).

### Patterns of antibiotic resistance

Antibiotic resistance patterns were assessed based on AST results, with samples categorized into eight types: sputum, blood, fluid (*e.g.*, tracheobronchial aspirates, cerebrospinal fluid, pleural fluid), catheter, tissue (skin, wound, and soft tissue specimens), urine, faeces, and unknown. Variables included isolation date, sample type, bacterial species, and resistance profiles. MDR strains were defined as resistance to at least one antibiotic in three or more antibiotic classes [17].

### Antibiotic susceptibility testing

ASTs were conducted using the VITEK2 Compact system at Phu Tho Hospital and the BD Phoenix 100 system at 175 Hospital, combined with disk diffusion, gradient diffusion, and broth microdilution methods at both sites [18, 19]. Both testing systems adhered to the Clinical and Laboratory Standards Institute (CLSI) 2020 guidelines [20] and Vietnam’s Ministry of Health protocol of the Ministry of Health of Vietnam (Decision No. 127/QD-BYT, 2019) under the Vietnam Antimicrobial Resistance Surveillance System [21].

Enzyme production, including extended-spectrum beta-lactamases (ESBL) and carbapenemases, was identified through biochemical assays in VITEK 2 and BD Phoenix system [22]. For *S. aureus*, resistance to oxacillin and cefoxitin was determined by minimum inhibitory concentration (MIC) with thresholds of >2 μg/mL for oxacillin and > 4 μg/mL for cefoxitin, following CLSI standards [20, 23] The methicillin-resistant *S. aureus* (MRSA) rate was calculated as the percentage of *S. aureus* isolates that were resistant to either oxacillin or cefoxitin out of the total number of *S. aureus* isolates tested for these antibiotics [24].

### Bacterial and antibiotic categories

Targeted bacteria included Acinetobacter species (*A. baumannii*, *Acinetobacter lwoffii, A. hemolyticus, Acinetobacter junii)*, Klebsiella species (*Klebsiella pneumoniae, Klebsiella oxytoca, Klebsiella aerogenes, K. ozaena*), *P. aeruginosa*, *E. coli*, and *S. aureus* as per American Type Culture Collection (ATCC) standards [25]. ASTs covered 25 antibiotic families according to WHO guidelines [26].

### Covariates

Covariates included demographic factors (age, sex, and location of residence) and clinical indicators (admission diagnoses coded by ICD-10, month, and year of test intake). These variables were chosen for their established links to AMR in ICU settings, which were explored as potential confounders in previous studies [9, 10].

### Statistical analysis

Data were cleaned, preprocessed, and analysed in Python 3.12, using packages like Pandas, Numpy, Statsmodels, Matplotlib, Seaborn, and Scikit-learn [27]. We analysed datasets separately to compare AMR patterns between hospitals, while using combined data for temporal trend analysis and assessing associations between clinical factors and AMR prevalence. For temporal analysis, we aggregated data by month and year of specimen collection (AST intake date) across both hospitals over the study period (2020–2022) to examine resistance trends over time. In addition, we conducted a separate analysis focused on COVID-19-specific trends by evaluating data from COVID-19 patients at 175 Hospital.

A supervised machine learning approach, specifically a logistic regression model with 1,000 bootstrap resamples, was used to assess demographic and clinical factors associated with AMR prevalence [27]. Potential confounders, including age, sex, admission diagnosis, residence location, and time of AST intake, were simultaneously included in the model to adjust for their effects.

Categorical variables (*e.g.*, year, month, location) were transformed into binary (0/1) formats using label and one-hot encoding, where 1 indicated the presence of a characteristic (*e.g.*, admission in 2020) and 0 its absence. To assess multicollinearity, we conducted variance inflation factor (VIF) analysis after removing redundant categories; all resulting VIF values were below five (range: 1.01–3.68), indicating no significant multicollinearity (Supplementary Table S2) [28]. Model coefficients and their 95% confidence intervals (CIs) were calculated using bootstrap resampling to ensure robust effect estimates [27].

## Results

### AMR prevalence and trends of five targeted bacteria among ICU patients

In Table 1, the ICU patients with positive bacterial culture had a mean age of 60 years (interquartile range – IQR from 50 to 70), with more men than women admitted at both hospitals. Notably, COVID-19 patients were exclusive to 175 Hospital. From 2020 to 2022, respiratory diseases (37.2%) and unclassified diseases (34.7%) were primary admission diagnoses at Phu Tho Hospital, while COVID-19 and respiratory diseases were predominant at 175 Hospital (25.1% and 28.7%, respectively). Infections with five targeted bacteria occurred in 83.7% of ICU patients in Phu Tho Hospital and 92.1% in 175 Hospital, with *Acinetobacter spp.* and *Klebsiella spp.* most prevalent. At 175 Hospital, the median hospital lengths and ICU stays were 13 days (IQR 8–22) and 11 days (IQR 6–17), respectively.

**Table 1.** Characteristics of ICU patients with positive bacterial culture results at Phu Tho Hospital (*n* = 600) and 175 Hospital (*n* = 696)

Characteristics	Phu Tho Hospital		175 Hospital	
Mean [SD]				
Age at admission (year)	60.0	*[17.6]*	59.5	*[15.8]*
N, %				
Men	440	*73.3*	450	*64.7*
Having health insurance	559	*93.2*	561	*80.6*
Year of admission				
*2020*	254	*42.3*	340	*48.9*
*2021*	196	*32.7*	207	*29.7*
*2022*	150	*25.0*	149	*21.4*
Place of residence				
*Phu Tho*	566	*94.3*	1	*0.1*
*Ho Chi Minh*	0	*0.0*	497	*71.4*
*Other provinces*	34	*5.7*	198	*28.5*
Primary diagnosis at admission by ICD–10				
*Respiratory (J00–J99)*	223	*37.2*	200	*28.7*
*COVID–19 (U07)*	0	*0.0*	175	*25.1*
*Abnormal and not classified (R00–R99)*	208	*34.7*	76	*10.9*
*Injury and poison (S00–T88)*	68	*11.3*	39	*5.6*
*Circulatory system (I00–I99)*	50	*8.3*	130	*18.7*
*Infection (A00–B99)*	33	*5.5*	111	*15.9*
*Digestion (K00–K95)*	14	*2.3*	21	*3.0*
*Genitourinary(N00–N99)*	9	*1.5*	11	*1.6*
*Neurology (G00–G99)*	5	*0.8*	8	*1.1*
Other diseases^a^	15	*2.7*	43	*6.2*
Number of patients having targeted bacteria				
*Acinetobacter spp*	225	*37.5*	339	*48.7*
*Klebsiella spp*	151	*25.2*	346	*49.7*
*Pseudomonas aeruginosa*	146	*24.3*	152	*21.8*
*Escherichia coli*	86	*14.3*	69	*9.9*
*Staphylococcus aureus*	30	*5.0*	70	*10.1*
Having at least one of the above	502	*83.7*	641	*92.1*
Median [IQR]				
Age at admission	61	*[48–73]*	61	*[49–70]*
Length of stay in hospital (days)	NA	*NA*	13	*[8–22]*
Length of stay in ICU (days)	NA	*NA*	11	*[6–17]*

a Other diseases included diseases of the musculoskeletal system; endocrine diseases; neoplasms; skin, blood, and immune mechanism; mental disorders; and external causes of morbidity.

ICU: Intensive Care Unit, COVID-19: Coronavirus Disease 2019; ICD-10: International Code of Disease – 10th version; IQR: Interquartile Range (the difference between quartile one and quartile three); NA: Data not available due to inconsistencies in the electronic medical records (EMRs) during the study period.

The five targeted bacteria (Table 2) accounted for 78.3% and 76.2% of all non-duplicated isolates in Phu Tho Hospital and 175 Hospital, respectively. In Phu Tho Hospital, Acinetobacter species *(Acinetobacter spp.)* were the most prevalent (27.5%), predominantly *A. baumannii*, followed by Klebsiella species (*Klebsiella spp.)* (18.8%) and *P. aeruginosa* (18.1%). *E. coli* and *S. aureus* represented 10.3% and 3.6%, respectively, of the isolates. At 175 Hospital, *Klebsiella spp.* (mainly *K. pneumoniae)* were dominated (28.0%), followed by *Acinetobacter spp.* (25.4%), primarily *A. baumannii.* Among 441 Klebsiella isolates, *K. aerogenes* (n = 11, 2.5%) appeared only at 175 Hospital, with one isolate from a COVID-19 patient. It exhibited high resistance to cephalosporins (*e.g.*, cefazolin 100%, ceftazidime 85.7%, ceftriaxone 72.7%) and carbapenems (imipenem/meropenem 80%) (Supplementary Table S3).

**Table 2.** Distribution of targeted bacteria isolated from ICU patients in two hospitals

	Phu Tho Hospital (*n* = 856)		175 Hospital (*n* = 1,576)	
	*n*	*%*	*n*	*%*
Acinetobacter species^a^	235	*27.5*	401	*25.4*
*Acinetobacter baumannii*	229	*26.8*	*380*	*24.1*
*Acinetobacter lwoffii*	3	*0.4*	0	*0.0*
*Acinetobacter hemolyticus*	2	*0.2*	1	*0.1*
*Acinetobacter junii*	1	*0.1*	0	*0.0*
*Acinetobacter spp*	0	*0.0*	20	*1.3*
Klebsiella species^b^	162	*18.8*	441	*28.0*
*Klebsiella pneumoniae*	155	*18.1*	397	*25.2*
*Klebsiella oxytoca*	6	*0.7*	1	*0.1*
*Klebsiella aerogenes*	0	*0.0*	11	*0.7*
*Klebsiella ozaenae*	0	*0.0*	6	*0.4*
*Klebsiella spp*	0	*0.0*	25	*1.6*
*Pseudomonas aeruginosa*	155	*18.1*	216	*13.7*
*Escherichia coli*	88	*10.3*	71	*4.5*
*Staphylococcus aureus*	31	*3.6*	72	*4.6*
Enterobacter species	26	*3.0*	16	*1.0*
Enterococcus species	19	*2.2*	42	*3.3*
*Burkholderia cepacia*	13	*1.5*	57	*3.6*
Proteus species	10	*1.2*	25	*1.6*
*Stenotrophomonas maltophilia*	9	*1.1*	33	*2.1*
Streptococcus spp	2	*0.2*	24	*1.5*
Other bacteria	107	*12.5*	178	*11.3*

a In this study, Acinetobacter *spp.* was used for all kinds of Acinetobacter species.

b In this study, Klebsiella *spp.* was used for all kinds of Klebsiella species.

ICU: Intensive Care Unit.

**Figure 1.**
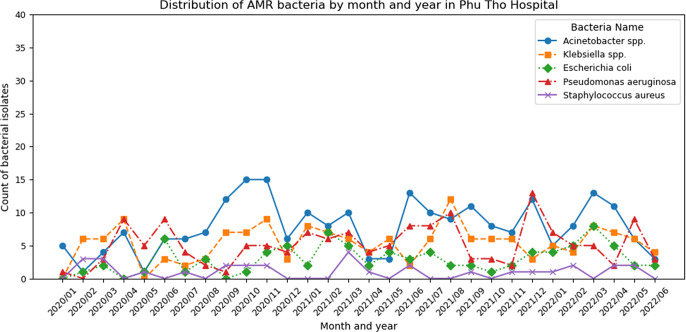
Number of antibiotic-susceptible test samples taken by month in Phu Tho Hospital.

**Figure 2.**
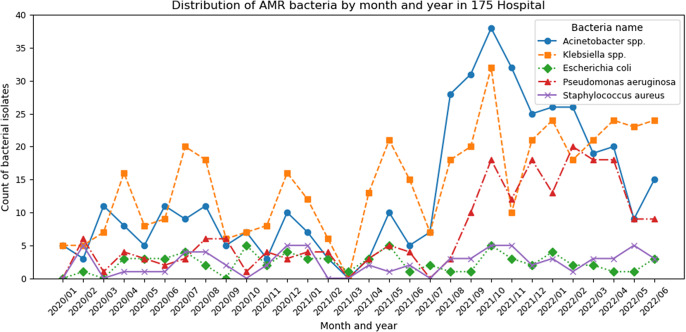
Number of antibiotic-susceptible test samples taken by month in 175 Hospital.

**Figure 3.**
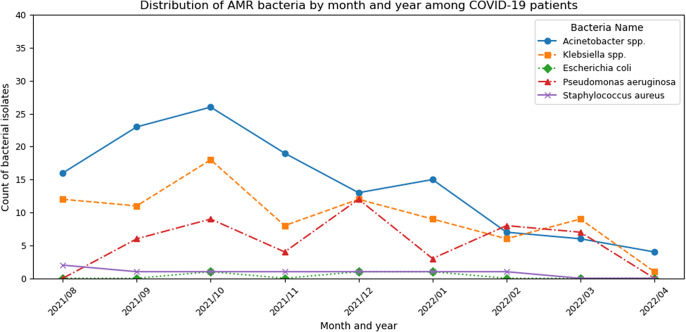
Number of isolates having AMR bacteria among COVID-19 patients in 175 Hospital.

For the monthly trends of bacterial isolates, Figures 1 and 2 shows that *Acinetobacter spp.* remained the most prevalent at Phu Tho Hospital, followed by *Klebsiella spp.* or *P. aeruginosa* (peaking in early 2020 and mid-2021). Meanwhile, *Klebsiella spp.* consistently dominated in 175 Hospital, except from July 2021 to March 2022, when *Acinetobacter spp.*’s prevalence reached the peak concurrently with the COVID-19 outbreaks in Ho Chi Minh City. During this period, *P. aeruginosa* cases doubled, while *E. coli* and *S. aureus* showed declining trends. Figure 3 highlights bacterial AMR among COVID-19 patients at 175 Hospital (August 2021 to April 2022) with *Acinetobacter spp.* peaking in October 2021 (26 isolates) and November 2021 (19 isolates). *Klebsiella spp.* shows the second highest number, peaking in October 2021 (18 isolates) and declining thereafter. *P. aeruginosa, E. coli, and S. aureus* remain low, with counts generally below five isolates per month, though *P. aeruginosa* shows a slight increase in December 2021 (12 isolates).

### Antibiotic resistance to different antibiotics and antibiotic families

Tables 3 and 4 illustrate the resistance to the common antibiotics used in AST. *Acinetobacter spp., Klebsiella spp.*, and *P. aeruginosa* exhibited high resistance rates (80–100%) to 90% of tested antibiotics, except for amikacin, colistin, trimethoprim-sulfamethoxazole, tobramycin, and doripenem, having resistance rates below 50%. The resistance of *E. coli* ranged from 60–80% in both hospitals. *S. aureus* had higher resistance rates in Phu Tho Hospital. In 175 Hospital, ESBL production was found in 35.2% of *E. coli* and 10.9% of *Klebsiella spp.* while carbapenemase production was found in 63.6% for *Acinetobacter spp.*, 63.9% for *Klebsiella spp.*, 52.9% for *P. aeruginosa*, and 16.9% for *E. coli.* Additionally, 66.5% of *S. aureus* were identified as MRSA. Both hospitals exhibited alarmingly high MDR rates, particularly elevated levels in 175 Hospital. At Phu Tho Hospital, MDR rates were 90.2% for *Acinetobacter spp.*, 85.1% for *Klebsiella spp.*, 87.7% for *P. aeruginosa*, 76.1% for *E. coli*, and 90.3% for *S. aureus.* 175 Hospital showed even higher rates for *Acinetobacter spp.* (95.8%) and *Klebsiella spp.* (90.0%) but lower MDR rates for *S. aureus* (51.4%).

**Table 3.** Antibiotic resistance rate of *Acinetobacter spp., Klebsiella spp., Pseudomonas aeruginosa, and Escherichia coli* towards the most commonly used antibiotics in the Antibiotic Susceptibility Test

Name of antibiotics and antibiotic families	Acinetobacter spp		Klebsiella spp		P. aeruginosa		E. coli	
	Phu Tho Hospital	175 Hospital	Phu Tho Hospital	175 Hospital	Phu Tho Hospital	175 Hospital	Phu Tho Hospital	175 Hospital
(%)								
Aminoglycosides	80.3	94.3	67.5	57.6	83.9	66.4	48.8	44.1
Amikacin	71.6	87.4	37.9	42.9	79.7	51.8	7.1	8.5
Gentamicin	73.1	93.6	56.4	58.2	81.2	63.6	45.2	48.3
Tobramycine	69.0	3.2	65.2	0.0	84.8	37.5	43.8	–
Beta-lactam	86.5	92.0	83.1	87.4	61.3	75.3	39.1	48.6
Amoxicillin-clavulanic acid	83.3	100.0	85.6	38.9	100.0	100.0	32.8	0.0
Ampicillin-sulbactam	68.7	89.2	79.5	89.9	100.0	100.0	53.3	52.6
Piperacillin-tazobactam	85.2	89.2	76.8	83.0	60.0	57.7	18.1	17.2
Carbapenems	79.2	95.7	75.5	82.9	84.3	86.8	13.8	24.3
Doripenem	80.0	**–**	73.3	12.5	85.5	–	12.5	**–**
Ertapenem	71.4	100	73.3	85.2	100	96.7	13.5	22.0
Imipenem	77.8	90.0	73.8	75.9	82.7	77.5	12.1	23.2
Meropenem	78.2	92.7	75.2	84.5	82.9	81.9	10.0	30.6
Cephalosporin first generation	–	100	–	92.0	0.0	100	–	82.1
Cefazolin	–	100	–	92.0	–	–	–	82.1
Cephalosporin second generation	90.0	100	88.1	84.0	100	100	72.7	38.9
Cefoxitin	88.9	100.0	81.0	84.4	100.0	100.0	35.3	41.7
Cefuroxime	88.9	100.0	89.8	88.0	100.0	100.0	75.0	42.9
Cephalosporin third generation	90.2	95.8	78.1	88.6	89.5	79.1	63.2	71.0
Cefotaxime	91.9	97.1	80.1	72.3	98.7	**–**	61.1	37.5
Ceftazidime	88.0	96.8	77.6	91.3	62.7	66.7	60.9	82.1
Ceftriaxone	89.7	87.5	72.5	86.1	88.9	100	58.6	69.1
Cephalosporin fourth generation	90.2	92.7	75.5	87.5	84.3	70.8	60.7	66.7
Cefepime	90.2	92.7	75.6	87.5	84.3	70.8	60.7	66.7
Fluoroquinolones	80.6	91.5	75.7	87.6	80.0	79.4	62.2	77.6
Ciprofloxacin	82.5	91.5	75.7	87.6	76.4	78.3	64.1	77.6
Levofloxacin	74.7	91.5	70.4	76.9	85.7	81.0	60.0	40.0
Minocycline	–	9.7	–	54.5	**–**	**–**	–	50.0
Piperacillin-Tazobactam	85.2	89.2	76.8	83.0	60.0	57.7	18.1	17.2
Glycylclines	**–**	31.0	**–**	9.1	**–**	91.8	**–**	1.8
Tigecycline	**–**	31.0	**–**	9.1	**–**	91.8	**–**	1.8
Monobactams	85.7	100	60.0	77.4	72.2	79.3	33.3	100
Aztreonam	85.7	100	60.0	77.4	72.2	79.3	33.3	100
Penicillin	100	98.8	99.1	99.2	100	97.2	83.3	94.6
Ampicillin	100.0	100.0	99.1	99.2	100.0	100.0	84.3	94.6
Phosphonics	**–**	77.8	62.0	68.0	**–**	100	10.8	16.7
Fosfomycin	**–**	77.8	62.0	68.0	**–**	100	10.8	16.7
Polymyxins	**–**	3.3	**–**	37.4	**–**	19.1	**–**	0.0
Colistin	**–**	3.3	**–**	37.4	**–**	19.1	**–**	0.0
Tetracyclines	42.6	9.7	70.8	55.6	66.7	97.2	75.0	50.0
Minocycline	**–**	9.7	**–**	54.5		97.2		50.0
Doxycycline	35.8	**–**	0.0	**–**	66.7	**–**	**–**	**–**
Tetracycline	48.0	**–**	73.9	**–**	**–**	**–**	75.0	**–**
Trimethoprim derivatives	70.6	81.4	55.4	77.5	100	93.9	68.9	70.9
Trimethoprim**–**sulfamethoxazole	70.6	81.4	55.7	77.5	100	93.9	68.9	70.9
ESBL	**–**	**–**	**–**	10.9	**–**	0.0	**–**	35.2
Carbapenemase	**–**	63.6	**–**	63.9	**–**	52.9	**–**	16.9
Multidrug resistance	90.2	95.8	85.1	90.0	87.5	83.8	76.1	76.1

ESBL: extended-spectrum beta-lactamase.

The symbol ‘–’ indicates that the test was not applicable or not performed for that particular antibiotic-bacteria combination.

**Table 4.** Antibiotic resistance rate of *Staphylococcus aureus* to the most commonly used antibiotics in the Antibiotic Susceptibility Test

Name of antibiotics and antibiotic families	Phu Tho Hospital	175 Hospital
Aminoglycosides	37.9	46.5
Gentamicin	37.9	46.5
Carbapenems	70.4	–
Ertapenem	69.2	–
Cephalosporin second generation	66.7	92.9
Cefoxitin	66.7	92.9
Fluoroquinolones	33.3	41.9
Ciprofloxacin	33.3	42.9
Levofloxacin	30.0	50.0
Moxifloxacin	29.6	50.0
Glycopeptides	6.7	0.0
Vancomycin	6.7	0.0
Teicoplanin	–	0.0
Lincosamides	82.1	60.0
Clindamycin	82.1	60.0
Lipopeptides	–	0.0
Daptomycin	–	0.0
Macrolides	84.3	58.1
Azithromycin	75.0	40.0
Erythromycin	85.7	58.1
Oxazolidinones	3.3	2.2
Linezolid	3.3	2.2
Penicillins	100	87.1
Ampicillin	100	100
Oxacillin	73.3	61.4
Penicillin G	100	98.2
Polimixins	–	70.1
Colistin	–	70.1
Tetracyclin	47.8	31.1
Doxycycline	0.0	0.0
Minocycline	–	0.0
Tetracycline	50.0	35.2
Trimethoprim deliveratives	34.5	32.1
Trimethoprim-sulfamethoxazole	34.5	32.1
Methicilin Resistant *S. aureus* (MRSA)	71.8	66.5
Multidrug resistance	90.3	51.4

The symbol ‘–’ indicates that the test was not applicable or not performed for that particular antibiotic-bacteria combination.

More detailed information on AMR bacteria categorized by cause of diagnosis admission could be found in Supplementary Table S1. In Phu Tho Hospital, *Acinetobacter spp.* were the most popular bacteria in patients with respiratory diseases (26.3% of positive cases), followed by *P. aeruginosa* (23.7%) and *Klebsiella spp.* (18.8%). Meanwhile, in 175 Hospital, *Acinetobacter spp.* were prevalent among COVID-19 patients (32.6% of cases), and *Klebsiella spp.* accounted for 21.8%.

### Factors associated with the prevalence of AMR bacteria

Table 5 shows the results of the logistic regression model with 1,000 times of bootstrap resampling. Men were less likely to have *E. coli* infections compared to women (coef = −0.73, 95% CI: [−1.12, −0.33]), and older patients had a lower likelihood of *S. aureus* infections than younger ones (coef = −0.30, 95% CI: [−0.55, −0.04]). COVID-19 patients showed a higher prevalence of *Acinetobacter spp.* compared to those without COVID-19 (coef = 0.46, 95% CI: [0.03, 0.89]), but a significantly lower prevalence of AMR *E. coli* (coef = −1.61, 95% CI: [−2.37, −0.85]) and *S. aureus* (coef = −1.04, 95% CI: [−1.84, −0.25]). Patients residing in Phu Tho had a lower prevalence of *Klebsiella spp.* (coef = −0.59, 95% CI: [−0.91, −0.26]) but higher rates of *Acinetobacter spp.* (coef = 0.40, 95% CI: [0.07, 0.73]) and *P. aeruginosa* (coef = 0.58, 95% CI: [0.18, 0.98]) compared to those from other regions. Regarding seasonality, patients admitted in May were less likely to develop AMR in *Acinetobacter spp.* (coef = −0.67, 95% CI: [−1.17, −0.17]) but more likely for *Klebsiella spp.* (coef = 0.46, 95% CI: [0.03, 0.88]) than those not admitted in this month. These associations highlight the complex interplay of patient demographics, admission timing, and sample types in understanding AMR prevalence.

**Table 5.** Associations between demographic and clinical indicators and the prevalence of AMR bacteria in both hospitals

Variables	Reference group (0)	Acinetobacter spp.		Klebsiella spp.		Pseudomonas aeruginosa		Escherichia coli		Staphylococcus aureus	
		coef	95% CI	coef	95% CI	coef	95% CI	coef	95% CI	coef	95% CI
Age at admission	*Continuous*	0.01	[−0.11, 0.14]	0.10	[−0.02, 0.22]	−0.06	[−0.21, 0.09]	0.16	[−0.06, 0.37]	−0.30	[−0.55, −0.04]
Sex	*Female*	0.08	[−0.17, 0.32]	0.26	[0.02, 0.50]	−0.08	[−0.37, 0.21]	−0.73	[−1.12, −0.33]	−0.01	[−0.49, 0.48]
Year of admission											
2020	*Not 2020*	−0.28	[−0.61, 0.06]	0.19	[−0.13, 0.51]	−0.29	[−0.66, 0.09]	0.91	[0.42, 1.41]	0.41	[−0.16, 0.99]
2021	*Not 2021*	−0.13	[−0.44, 0.17]	0.17	[−0.12, 0.45]	−0.01	[−0.36, 0.33]	0.64	[0.17, 1.12]	0.02	[−0.55, 0.59]
Residential location											
Ho Chi Minh City	*Not Ho Chi Minh City*	0.07	[−0.23, 0.38]	−0.01	[−0.30, 0.28]	0.04	[−0.33, 0.42]	−0.37	[−0.90, 0.17]	0.10	[−0.48, 0.68]
Phu Tho province	*Not Phu Tho province*	0.40	[0.07, 0.73]	−0.59	[−0.91, −0.26]	0.58	[0.18, 0.98]	0.25	[−0.29, 0.80]	−0.63	[−1.26, 0.01]
Diagnosis at admission by ICD–10											
Having respiratory diseases [J00-J99]	*No respiratory diseases*	0.16	[−0.23, 0.55]	−0.18	[−0.56, 0.19]	0.69	[0.28, 1.11]	−0.48	[−1.04, 0.09]	−0.45	[−1.03, 0.14]
Having abnormal symptoms in unclassified diseases [R00-R99]	*No abnormal symptoms in unclassified diseases*	−0.05	[−0.49, 0.39]	−0.17	[−0.60, 0.25]	0.37	[−0.12, 0.85]	0.43	[−0.13, 1.00]	−0.11	[−0.79, 0.56]
COVID19 (U07)	*No COVID19*	0.46	[0.03, 0.89]	−0.23	[−0.67, 0.22]	0.41	[−0.11, 0.94]	−1.61	[−2.37, −0.85]	−1.04	[−1.84, −0.25]
Having circulatory system diseases [I00-I99]	*No circulatory system diseases*	0.08	[−0.35, 0.51]	0.06	[−0.35, 0.47]	0.39	[−0.12, 0.89]	0.08	[−0.56, 0.71]	0.10	[−0.56, 0.76]
Having infectious diseases [A00-B99]	*No infectious diseases*	0.05	[−0.42, 0.51]	−0.06	[−0.52, 0.40]	0.48	[−0.07, 1.03]	0.21	[−0.44, 0.85]	−0.13	[−0.83, 0.57]
Injury and poison (S00-T88)	*No injury and poison*	0.48	[−0.03, 0.99]	−0.07	[−0.57, 0.44]	0.31	[−0.25, 0.87]	−0.55	[−1.35, 0.24]	−0.40	[−1.21, 0.40]
Sample types											
Blood	*Not Blood*	−0.95	[−1.33, −0.58]	−0.39	[−0.77, −0.02]	−1.34	[−1.84, −0.84]	1.37	[0.91, 1.83]	1.12	[0.60, 1.63]
Catheter	*Not Catheter*	−0.72	[−1.47, 0.03]	0.02	[−0.68, 0.72]	−0.54	[−1.33, 0.25]	0.03	[−1.00, 1.05]	−0.14	[−1.06, 0.78]
Faeces	*Not Faeces*	−1.02	[−1.49, −0.56]	−0.02	[−1.02, 0.97]	−0.67	[−1.07, −0.28]	0.65	[−0.71, 2.00]	−0.17	[−0.32, −0.02]
Fluid	*Not Fluid*	−0.21	[−0.61, 0.18]	−0.09	[−0.50, 0.32]	−0.41	[−0.91, 0.10]	1.60	[0.91, 2.28]	−0.16	[−0.96, 0.65]
Tissue	*Not Tissue*	−0.30	[−0.72, 0.12]	−0.33	[−0.75, 0.10]	−0.41	[−0.96, 0.15]	1.29	[0.58, 2.01]	0.33	[−0.48, 1.14]
Urine	*Not Urine*	−1.98	[−2.60, −1.37]	−0.01	[−0.53, 0.51]	−0.70	[−1.32, −0.07]	2.11	[1.55, 2.68]	−0.98	[−1.19, −0.77]
Pus	*Not Pus*	−0.63	[−1.52, 0.26]	0.56	[−0.47, 1.59]	−0.94	[−1.36, −0.51]	−0.32	[−0.52, −0.11]	2.18	[0.82, 3.54]
Others	*Not Others*	−0.27	[−0.76, 0.23]	−0.08	[−0.24, 0.08]	−0.15	[−0.47, 0.16]	−0.05	[−0.16, 0.06]	−0.01	[−0.04, 0.02]
Month of admission											
January	*Not January*	−0.05	[−0.49, 0.39]	−0.05	[−0.50, 0.39]	−0.36	[−0.87, 0.15]	−0.21	[−0.86, 0.45]	−0.12	[−0.85, 0.62]
February	*Not February*	−0.30	[−0.76, 0.15]	−0.03	[−0.51, 0.44]	0.14	[−0.34, 0.62]	0.09	[−0.52, 0.70]	0.10	[−0.63, 0.83]
March	*Not March*	−0.13	[−0.58, 0.32]	−0.08	[−0.54, 0.38]	−0.18	[−0.66, 0.30]	0.54	[−0.06, 1.15]	0.15	[−0.57, 0.86]
April	*Not April*	−0.20	[−0.67, 0.26]	0.25	[−0.19, 0.69]	−0.10	[−0.62, 0.41]	0.22	[−0.45, 0.89]	−0.17	[−0.90, 0.56]
May	*Not May*	−0.67	[−1.17, −0.17]	0.46	[0.03, 0.88]	−0.05	[−0.56, 0.45]	0.31	[−0.31, 0.92]	0.07	[−0.58, 0.72]
June	*Not June*	−0.10	[−0.53, 0.32]	0.03	[−0.38, 0.45]	−0.09	[−0.57, 0.39]	0.38	[−0.23, 0.99]	−0.06	[−0.76, 0.63]
July	*Not July*	0.10	[−0.39, 0.59]	0.08	[−0.41, 0.56]	−0.53	[−1.14, 0.08]	0.07	[−0.67, 0.80]	−0.46	[−1.27, 0.35]
August	*Not August*	0.19	[−0.27, 0.64]	0.18	[−0.29, 0.65]	−0.16	[−0.68, 0.37]	−0.64	[−1.42, 0.14]	−0.71	[−1.52, 0.11]
September	*Not September*	0.35	[−0.09, 0.78]	0.05	[−0.41, 0.51]	−0.11	[−0.68, 0.46]	−1.05	[−1.78, −0.32]	−0.15	[−0.93, 0.62]
October	*Not October*	0.31	[−0.12, 0.73]	0.06	[−0.36, 0.49]	−0.15	[−0.69, 0.40]	−0.21	[−0.95, 0.53]	−0.37	[−1.15, 0.41]
November	*Not November*	−0.05	[−0.49, 0.39]	−0.05	[−0.50, 0.39]	−0.36	[−0.87, 0.15]	−0.21	[−0.86, 0.45]	−0.12	[−0.85, 0.62]

AMR: Antimicrobial resistance; coef: coefficient; 95% CI: 95% confidence interval; ICD-10: International Code Diseases – 10th version; COVID-19: coronavirus disease 2019.

## Discussion

This study assessed the prevalence and risk factors for AMR associated with the five key bacteria among ICU patients at Phu Tho Hospital and 175 Hospital between January 2020 and June 2022. Results revealed a high prevalence of AMR and MDR, particularly in *Acinetobacter spp., Klebsiella spp.*, and *P. aeruginosa. Acinetobacter spp.* predominated in Phu Tho Hospital, while *Klebsiella spp.* led in 175 Hospital, where COVID-19 patients accounted for 70.1% of cases involving these bacteria. Bootstrap logistic regression analysis identified significant associations between AMR prevalence and several factors, including patient demographics (age, sex, residential location), clinical characteristics (sample type, admission diagnosis), and month of bacterial isolation.

### The prevalence of AMR in targeted bacteria

In this study, the predominance of *Acinetobacter spp., Klebsiella spp.*, and *P. aeruginosa* (65%–67% of all isolated samples) aligned with studies from Vietnam in 2020 [6] and other LMICs in 2021 [2], particularly the exceptionally high carbapenem resistance among *Klebsiella spp.* and *P. aeruginosa. P. aeruginosa* ranked as the second-highest infection in respiratory cases at Phu Tho, mirroring its role in Southeast Asian ICUs after *A. baumannii* [29]. High carbapenem resistance in *P. aeruginosa* (75.5% at Phu Tho, 82.9% at 175 Hospitals) far exceeded national surveillance rates from 2020 (45.3%) [6].

Interestingly, the higher prevalence of *Acinetobacter spp.* compared to *Klebsiella spp.* in Phu Tho Hospital for the whole period were similar to a hospital ICU data in Ho Chi Minh city in 2015 (before COVID-19) [30]. Meanwhile, *Klebsiella spp.* predominated at 175 Hospital, except during the COVID-19 outbreak in Ho Chi Minh city (July 2021–January 2022), when *Acinetobacter spp.* increased significantly and ranked highest, which implied pandemic-related AMR selection pressures. Alarmingly, colistin resistance in *Acinetobacter spp.* at 175 Hospital (3.3%) doubled compared to 2015 levels (1.5%), signalling the reduced efficacy of this last-resort antibiotic [30].

*Klebsiella spp.*’s high resistance to carbapenem and third-generation cephalosporins reflected the similar trends in LMICs, such as China, India, Pakistan, Egypt, and Vietnam [2] and the opposing downward trend in the United States and Europe [31]. Rising carbapenem resistance among *Klebsiella spp.* in Vietnam and in LMICs may be linked to carbapenemase production of *K. pneumoniae* [32] and natural ampC enzyme overexpression in *K. aerogenes* [33]. Risk factors for high AMR rates of *Acinetobacter spp.* and *Klebsiella spp.* in ICUs likely included ventilator-associated pneumonia (VAP) (due to high ventilator bed occupancy, particularly for COVID-19 patients [34]), invasive procedures (*e.g.*, urinary catheterization), prolonged hospitalization, weakened immune and microbiome systems [2, 35], as well as the misuse of broad-spectrum antibiotics such as cephalosporins and penicillins [36]. MDR rates for *Acinetobacter spp.* and *Klebsiella spp.* surpassed rates from a national study 10 years ago [37], likely due to overuse of cephalosporins and quinolones, prophylactic antibiotic misuse, and patient overcrowding, healthcare workers’ habit of receiving broad-spectrum antibiotics, or incomplete sterilization of the material [38].

While this study focused on ICU patients due to their heightened AMR risk, recent literature highlights remarkable differences between ICU and non-ICU settings in Vietnam during the COVID-19 pandemic. For example, a study from a Hanoi tertiary hospital (2014–2021) reported the MRSA rate of 50% in ICU *versus* (*vs.*) 33.3% in non-ICU, *Acinetobacter spp.* meropenem resistance 88.0%–91.0% in ICU *vs.* 56.0%–58.0% in non-ICU, *K. pneumoniae* carbapenem resistance (6%–59% in ICU *vs.* 13.0%–16.0% in non-ICU, *P. aeruginosa* meropenem resistance 75.0% *vs.* 55.0% [39], and *E. coli* cefotaxime resistance 62.0% *vs.* 49.0% [40]. National 2020 data found higher rates for MRSA (78%) and *E. coli* ceftriaxone resistance (67.9%) but lower for carbapenem resistance (*e.g.*, 45.3% for *P. aeruginosa*, 87.8% for *Acinetobacter spp.*) [21]. Increased ICU antibiotic use, especially carbapenems [39–41], and rising community-acquired resistance (*e.g.*, fluoroquinolones, cephalosporins) [41] likely increased AMR’s pervasiveness across hospital settings. These trends underscore the need for expanded AMR surveillance across both ICU and non-ICU settings, in line with national recommendations [21].

### Factors associated with the different prevalence of antibiotic-resistant bacteria

The observed regional differences in antimicrobial resistance (AMR) rates between 175 Hospital (Southern Vietnam) and Phu Tho Hospital (Northern Vietnam) reflect a complex interplay of patient demographics, antibiotic prescribing practices, infection control measures, and hospital-specific roles during the COVID-19 pandemic, as well as regional variations in antibiotic use outside hospital settings. Environmental factors, such as seasonality, may also contribute to the sporadic occurrence of AMR bacteria.

Patient demographics (*e.g.*, age and sex) and clinical characteristics (diagnosis) are significantly associated with the prevalence of AMR bacteria. Notably, AMR *E. coli* and *S. aureus* were less likely to occur among higher age, female, and COVID-19 patients, that may reflect the unique local patient demographics, as mentioned in some studies in global and in Europe [1, 42]. Geographically, Northern Vietnam (Phu Tho) showed higher *Acinetobacter spp.* and lower AMR *Klebsiella spp.* rates than the South (175 Hospital), consistent with prior Vietnamese studies highlighting regional and temporal AMR variations [7, 43].

Antibiotic prescribing practices and hospital roles during COVID-19 further shaped AMR patterns. As a major COVID-19 treatment centre in urban Ho Chi Minh City, 175 Hospital faced elevated AMR rates, likely due to intensified broad-spectrum antibiotic use (*e.g.*, carbapenems) for critically ill patients during the fourth wave (April 2021–March 2022) [44, 45] which already increased the selective pressure and resistance likely increased, fostering resistant strains. High patient volumes and resource strain may have also weakened infection control, amplifying AMR spread. Conversely, Phu Tho Hospital, a rural facility not treating COVID-19, had lower AMR rates, linked to reduced antibiotic exposure and less severe cases (10 deaths among 5,500 COVID-19 cases). Conversely, Phu Tho Hospital, a rural facility not treating COVID-19, had lower AMR rates, possibly due to reduced antibiotic exposure and less severe cases (10 deaths among 5,500 COVID-19 cases) [46]. However, its higher MRSA rates compared to 175 Hospital suggest gaps in infection prevention and control (IPC), particularly in the ICU, where HAIs may have contributed [39].

Urban settings like Ho Chi Minh City exhibited higher AMR due to dense populations, healthcare pressures, and easier antibiotic access *via* pharmacies, often without prescriptions, that led to AMR spread in community settings [47]. Though both hospitals are public ones and follow national IPC guidelines [44], but the effectiveness of these measures in 175 Hospital was likely compromised by patient overload and resource constraints during the pandemic [31]. For VAP, standard measures were implemented at both sites [44]. However, 175 Hospital, with more ventilated COVID-19 patients, may have experienced higher VAP rates due to resource limitations, reflecting pre-pandemic trends in Vietnamese central hospitals where Acinetobacter, Klebsiella, and *P. aeruginosa* were predominant [7, 48]. Besides, the antibiotic stewardship programs (*e.g.*, antibiotic prescription committees, usage monitoring) were in place at both hospitals, but 175 Hospital struggled more with broader-spectrum antibiotic use, a pattern also observed in other Western Pacific and Southeast Asian countries [43].

Seasonal factors also influenced AMR, with lower *Acinetobacter spp.* rates in May, possibly tied to climate variations like humidity, which possibly affected the spread and resistance of respiratory pathogens [29]. These findings emphasize the importance of integrating patient, hospital, regional, and environmental factors into AMR surveillance and control strategies in Vietnam.

## Strengths and limitations

This study is among the few in Vietnam and Southeast Asia that utilized EMR to examine the prevalence of AMR and identify associated factors. However, several limitations must be acknowledged. *First*, the retrospective design and pandemic context may have introduced some kinds of bias. Selection bias may arise from milder cases avoiding care, information bias from inconsistent COVID-19 testing accuracy, and unmeasured confounding from undocumented comorbidities or non-ICU antibiotic use (*e.g.*, outpatient or other ward departments). Reliance on EMRs risks misclassification due to incomplete antibiotic histories, and the inability to distinguish community- from hospital-acquired infections restricts insight into nosocomial spread.

*Second*, methodological constraints include inter-hospital variability in laboratory protocols and the lack of reference laboratory confirmation for AST results [49], potentially underestimating resistance rates. Uneven sampling and incomplete testing of antibiotic families across patients and study sites may further skew resistance rates. Furthermore, the COVID-19 pandemic likely influenced the sampling strategy, with clinicians potentially prioritizing COVID-19 patients for testing. Besides, limited resources and capability in Vietnam’s laboratories during and even before the pandemic – where AST is primarily performed for severe cases at provincial/central hospitals [38, 45] – likely led to over-sampling of resistant infections, potentially inflating AMR rates.

*Finally*, unmeasured factors like residency patterns, microbiome influences, and dietary antibiotic exposure [35], could further affect AMR patterns. While these limitations are typical of retrospective EMR studies, they highlight the needs for future prospective designs with standardized data collection.

## Conclusion

This study of 2,432 electronic medical records from 1,296 patients at 175 Hospital and Phu Tho Hospital revealed distinct AMR patterns in ICUs: *Acinetobacter spp.* predominated at Phu Tho Hospital, while Klebsiella *spp.* were most prevalent at 175 Hospital, with resistance rates for cephalosporins, carbapenems, and fluoroquinolones ranging from 75% to 100%, and MDR rates at 83.8%–95.8%. This study also highlights the associations between AMR prevalence in Vietnam ICUs and local factors, including patient demographics (age, sex), clinical characteristics (sample types, comorbidities), seasonality, and COVID-19 admissions. The findings underscore the need for tailored antibiotic stewardship programmes, robust infection control practices, and continuous regional surveillance, particularly in high-risk settings like ICUs, to address such disparities effectively. Future research should explore how community-level resistance, potentially elevated during the pandemic, influences hospital AMR rates across these regions.

## Supporting information

10.1017/S0950268825100307.sm001 Do et al. supplementary material Do et al. supplementary material

## Data Availability

The data are not publicly available due to sensitivity reasons but are available upon reasonable request to the corresponding author (dda.icd@gmail.com).

## Abbreviations

*A. baumannii*: *A. baumannii*
AI: Artificial Intelligence
AMR: Antimicrobial resistance
AST: Antimicrobial Susceptibility Testing
ATCC: American Type Culture Collection
BD Phoenix: Becton Dickinson Phoenix
CI: Confidence interval
CLSI: Laboratory Standards Institute
coef: Coefficient
COVID-19: Coronavirus Disease 2019
*E. coli*: *E. coli*
EMR: Electronic medical record
ESBL: Extended-spectrum beta-lactamase
GDP: Gross domestic product
GLASS: Global Antimicrobial Resistance Surveillance
HAI: Hospital-acquired infections
HICs: High-income countries
ICD-10: International Code of Diseases – 10th version
ICUs: Intensive Care Units
IQR: Interquartile Range
LMICs: Low- and middle-income countries
MDR: Multidrug resistance
ML: Machine learning
MRSA: Methicillin-resistant *S. aureus*
*P. aeruginosa*: *P. aeruginosa*
*S. aureus*: *S. aureus*
SD: Standard deviation
USD: US Dollar
VAP: Ventilator-associated pneumonia
VIF: Variance Inflation Factor
WHO: World Health Organization

## Supplementary material

The supplementary material for this article can be found at http://doi.org/10.1017/S0950268825100307.
