# Supplementary material for: Antibiotic resistance among ICU patients during the COVID-19 pandemic and its associated factors: a retrospective study using electronic medical records in two Vietnamese hospitals
Source: Epidemiol Infect. 2025 Jul 17;153:e93. doi: 10.1017/S0950268825100307 (PMC12394013; doi:10.1017/S0950268825100307)
Supplement: Do et al. supplementary material [file S0950268825100307sup001.docx]

**Supplementary Table S1.** Distribution of bacteria by diagnosis caused by the ICD-10 in two hospitals

|  | **All types of bacteria** | | | **Acinetobacter spp** | | **Klebsiella spp** | | ***Pseudomonas aeruginosa*** | | ***Escherichia coli*** | | ***Staphylococcus aureus*** | |
| --- | --- | --- | --- | --- | --- | --- | --- | --- | --- | --- | --- | --- | --- |
|  | n | % | n | | % | n | % | n | % | n | % | n | % |
| ***Phu Tho Hospital*** | **856** | ***100*** | **235** | | ***100*** | **162** | ***100*** | **155** | ***100*** | **88** | ***100*** | **31** | ***100*** |
| Respiratory (J00-J99) | 308 | *35.9* | 81 | | *34.5* | 58 | *36.0* | 73 | *47.1* | 25 | *28.4* | 7 | *22.6* |
| Abnormal and not classified (R00-R99) | 288 | *33.6* | 69 | | *29.4* | 55 | *34.2* | 46 | *29.7* | 44 | *50.0* | 9 | *29.0* |
| Injury and poison (S00- T88) | 101 | *11.8* | 34 | | *14.5* | 24 | *14.9* | 17 | *11.0* | 3 | *3.4* | 2 | *6.5* |
| Circulatory system (I00-I99) | 69 | *8.1* | 24 | | *10.2* | 11 | *6.8* | 12 | *7.7* | 6 | *6.8* | 3 | *9.7* |
| Infection (A00-B99) | 40 | *4.7* | 13 | | *5.5* | 5 | *3.1* | 5 | *3.2* | 2 | *2.3* | 6 | *19.4* |
| Digestion (K00-K95) | 18 | *2.1* | 3 | | *1.3* | 4 | *2.5* | 0 | *0.0* | 4 | *4.5* | 3 | *9.7* |
| Genitourinary (N00-N99) | 11 | *1.3* | 3 | | *1.3* | 0 | *0.0* | 1 | *0.6* | 2 | *2.3* | 0 | *0.0* |
| Metabolism (E00-E89) | 7 | *0.8* | 2 | | *0.9* | 1 | *0.6* | 0 | *0.0* | 1 | *1.1* | 1 | *3.2* |
| Neurology (G00-G99) | 6 | *0.7* | 2 | | *0.9* | 2 | *1.2* | 0 | *0.0* | 0 | *0.0* | 0 | *0.0* |
| Neoplasms (C00-D49) | 4 | *0.5* | 3 | | *1.3* | 0 | *0.0* | 1 | *0.6* | 0 | *0.0* | 0 | *0.0* |
| Musculoskeletal (M00-M99) | 3 | *0.4* | 1 | | *0.4* | 1 | *0.6* | 0 | *0.0* | 1 | *1.1* | 0 | *0.0* |
| Other diseases* | 1 | *0.1* | 0 | | *0.0* | 1 | *0.6* | 0 | *0.0* | 0 | *0.0* | 0 | *0.0* |
|  | n | % | n | | % | n | % | n | % | n | % | n | % |
| ***175 Hospital*** | **1576** | ***100*** | **401** | | ***100*** | **441** | ***100*** | **216** | ***100*** | **71** | ***100*** | **72** | ***100*** |
| Respiratory (J00-J99) | 425 | 27.0 | 109 | | *37.3* | 113 | *34.5* | 75 | *53.2* | 12 | *20.3* | 17 | *30.9* |
| COVID-19 (U07) | 395 | 25.1 | 129 | | *44.2* | 86 | *26.2* | 51 | *36.2* | 3 | *5.1* | 8 | *14.5* |
| Circulatory system (I00-I99) | 244 | 15.5 | 57 | | *19.5* | 84 | *25.6* | 28 | *19.9* | 17 | *28.8* | 16 | *29.1* |
| Infection (A00-B99) | 203 | 12.9 | 40 | | *13.7* | 63 | *19.2* | 27 | *19.1* | 23 | *39.0* | 6 | *10.9* |
| Abnormal and not classified (R00-R99) | 107 | 6.8 | 21 | | *7.2* | 34 | *10.4* | 12 | *8.5* | 6 | *10.2* | 7 | *12.7* |
| Injury and poison (S00-T88) | 69 | 4.4 | 23 | | *7.9* | 19 | *5.8* | 7 | *5.0* | 1 | *1.7* | 5 | *9.1* |
| Metabolism (E00-E89) | 32 | 2.0 | 4 | | *1.4* | 10 | *3.0* | 5 | *3.5* | 2 | *3.4* | 2 | *3.6* |
| Digestion (K00-K95) | 31 | 2.0 | 8 | | *2.7* | 12 | *3.7* | 1 | *0.7* | 2 | *3.4* | 1 | *1.8* |
| Neoplasms (C00-D49) | 14 | 0.9 | 3 | | *1.0* | 3 | *0.9* | 3 | *2.1* | 1 | *1.7* | 1 | *1.8* |
| Genitourinary(N00-N99) | 13 | 0.8 | 2 | | *0.7* | 2 | *0.6* | 4 | *2.8* | 2 | *3.4* | 1 | *1.8* |
| Musculoskeletal (M00-M99) | 11 | 0.7 | 0 | | *0.0* | 0 | *0.0* | 2 | *1.4* | 1 | *1.7* | 6 | *10.9* |
| Skin and tissue (L00-L99) | 11 | 0.7 | 3 | | *1.0* | 4 | *1.2* | 1 | *0.7* | 0 | *0.0* | 1 | *1.8* |
| Neurology (G00-G99) | 10 | 0.6 | 1 | | *0.3* | 6 | *1.8* | 0 | *0.0* | 0 | *0.0* | 1 | *1.8* |
| Other diseases** | 11 | 0.7 | 1 | | *0.3* | 5 | *1.5* | 0 | *0.0* | 1 | *1.7* | 0 | *0.0* |

*Other diseases included diseases of mental disorders.

** Other diseases included diseases of the blood and immune mechanism, mental disorders, and external causes of morbidity.

COVID-19: Coronavirus disease 2019; ICD-10: International Code of Disease – 10th version

**Supplementary Table S2:** Values of Variance Inflation Factor (VIF) to check the multicollinearity of variables

| **Variable** | **VIF** |
| --- | --- |
| ***Age at admission*** | ***1.24*** |
| ***Sex*** | ***3.12*** |
| ***Year of admission*** |  |
| 2020 | 2.58 |
| 2021 | 2.88 |
| ***Residential location*** |  |
| Ho Chi Minh City | 3.26 |
| Phu Tho province | 2.84 |
| ***Diagnosis at admission by ICD-10*** |  |
| Having respiratory diseases [J00-J99] | 3.68 |
| Having abnormal symptoms in unclassified diseases [R00-R99] | 2.71 |
| COVID19 (U07) | 2.65 |
| Having circulatory system diseases [I00-I99] | 2.28 |
| Having infectious diseases [A00-B99] | 1.97 |
| Injury and poison (S00-T88) | 1.92 |
| ***Sample types*** |  |
| Blood | 1.27 |
| Catheter | 1.04 |
| Feces | 1.03 |
| Fluid | 1.25 |
| Tissue | 1.21 |
| Urine | 1.09 |
| Pus | 1.03 |
| Others | 1.01 |
| ***Month of admission*** |  |
| January | 1.78 |
| February | 1.81 |
| March | 1.87 |
| April | 1.85 |
| May | 1.72 |
| June | 1.80 |
| July | 1.63 |
| August | 1.63 |
| September | 1.56 |
| October | 1.75 |
| November | 1.62 |

**Supplementary Table S3.** Antimicrobial resistance pattern of *Klebsiella aerogenes* in 175 Hospital

| Antibiotic | Number of Samples | Number of Resistant | Resistance Rate (%) |
| --- | --- | --- | --- |
| Amikacin | 10 | 1 | *10.0* |
| Amoxicillin/ Acid Clavunic | 1 | 0 | *0.0* |
| Ampicillin | 4 | 4 | *100.0* |
| Ampicillin-Sulbactam | 8 | 8 | *100.0* |
| Aztreonam | 4 | 1 | *25.0* |
| Cefazolin | 10 | 10 | *100.0* |
| Cefepime | 10 | 7 | *70.0* |
| Cefotaxime | 1 | 0 | *0.0* |
| Cefotaxime/ Acid Clavunic | 1 | 0 | *0.0* |
| Cefoxitin | 7 | 7 | *100.0* |
| Ceftazidim/ Acid Clavunic | 1 | 1 | *100.0* |
| Ceftazidime | 7 | 6 | *85.7* |
| Ceftazidime - avibactam | 2 | 2 | *100.0* |
| Ceftriaxone | 11 | 8 | *72.7* |
| Cefuroxime | 1 | 1 | *100.0* |
| Ciprofloxacin | 11 | 8 | *72.7* |
| Ertapenem | 9 | 7 | *77.8* |
| Fosfomycin | 2 | 2 | *100.0* |
| Gentamicin | 10 | 6 | *60.0* |
| Imipenem | 10 | 8 | *80.0* |
| Levofloxacin | 4 | 1 | *25.0* |
| Meropenem | 10 | 8 | *80.0* |
| Nitrofurantoin | 1 | 1 | *100.0* |
| Piperacillin-Tazobactam | 10 | 8 | *80.0* |
| Trimethoprim-sulfamethoxazole | 9 | 6 | *66.7* |


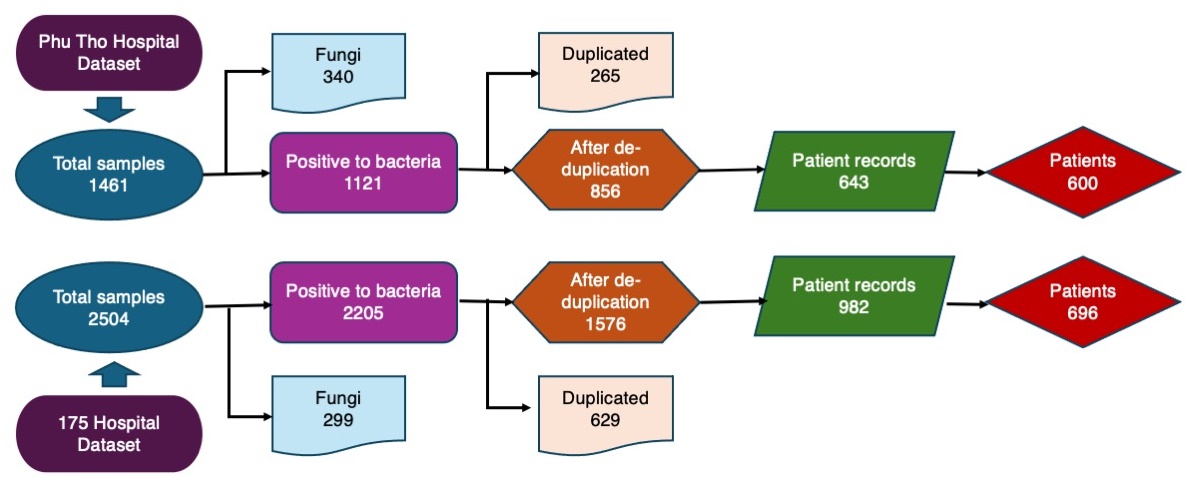


**Supplementary Figure S1.** Data screening process


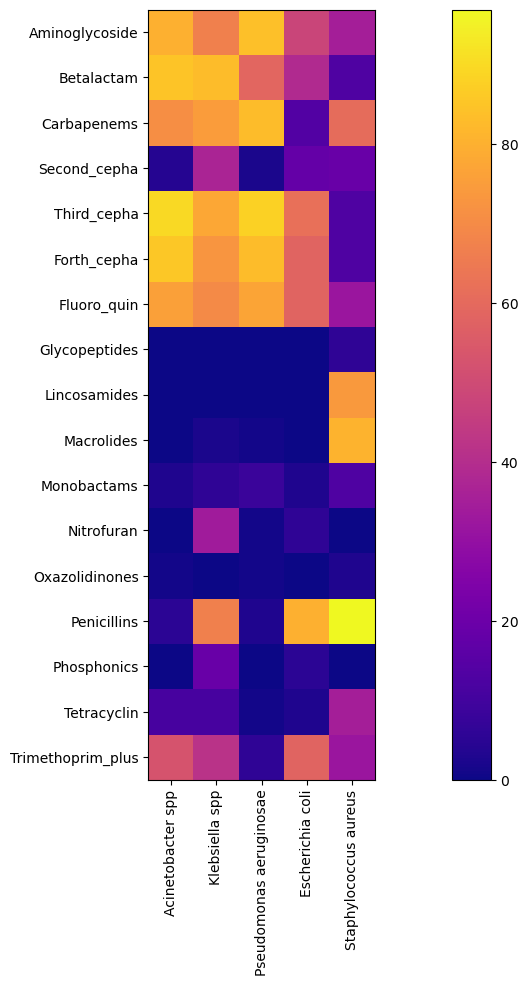


First_cepha: First-generation cephalosporin; Second_cepha: Second-generation cephalosporin; Third_cepha: Third-generation cephalosporin; Fourth_cepha: Fourth-generation cephalosporin; Trimethoprim_plus: Trimethoprim derivatives

**Supplementary Figure S2.** The resistance of each kind of bacteria to different antibiotic families in Phu Tho Hospital


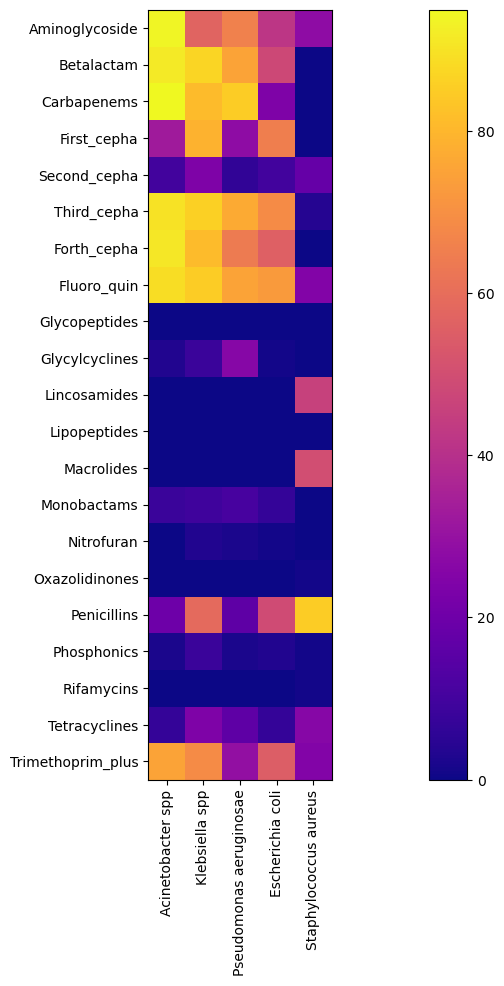


First_cepha: First-generation cephalosporin; Second_cepha: Second-generation cephalosporin; Third_cepha: Third-generation cephalosporin; Fourth_cepha: Fourth-generation cephalosporin; Trimethoprim_plus: Trimethoprim derivatives

**Supplementary Figure S3.** The resistance of each kind of bacteria to different antibiotic families in 175 Hospital
